# The effect of heatwaves on the number of visits to national parks and reserves

**DOI:** 10.1371/journal.pone.0289201

**Published:** 2023-08-09

**Authors:** Zohar Barnett-Itzhaki, Aviv Sar-Shalom, Liav Cohn, Lior Chen, Ofer Steinitz

**Affiliations:** 1 Faculty of Engineering, Ruppin Academic Center, Emek Hefer, Israel; 2 Research Group in Environmental and Social Sustainability, Ruppin Academic Center, Emek Hefer, Israel; 3 Ruppin Academic Center, Emek Hefer, Israel; 4 Israel Nature and Parks Authority, Jerusalem, Israel; Tribhuvan University, NEPAL

## Abstract

**Background:**

Climate change is leading to an increase in the frequency and intensity of heatwaves in many regions of the world. Climate change is also related to air pollution. Both heatwaves and air pollution have adverse health effects, and can also affect social behaviors, including tourism and touristic activities. The aim of this study was to examine the association between heatwaves, air pollution and visits to national parks and reserves in Israel.

**Methods:**

Data on 68,518 visits in 51 national parks and reserves in Israel in the years 2016–2019 was crossed with temperature and air pollution data (represented by particulate matter PM_10_) and analyzed using statistical tests.

**Results:**

Number of visits, as a function of temperature followed a unimodal distribution, in which more visits were reported on mild temperature days (in comparison to hot or cold days). In addition, the number of visits in sites with beaches was linearly correlated with temperature. Negative associations were found between number of visits and heatwaves, and between number of visits and exceedances in PM_10_ levels.

**Conclusions:**

Heatwaves were shown to have a negative effect on the number of visits in national parks and reserves in Israel. The negative association between exceedances in air pollution and number of visits may be mediated by the positive correlation between air pollution exceedance events and heatwaves.

## Background

The vulnerability of protected areas to climate change has been globally recognized, and many international efforts have addressed and are continuing to emphasize the urgent need to develop strategic adaptations to these changes. Climate changes affect not only the natural flora and fauna within parks and protected areas, but also tourism and touristic activities. In general, tourism is known as a highly climate-sensitive sector, that is significantly influenced by environmental changes [1]. For example, in countries with cold climates, the increase in outdoor temperature leads to opportunities for warm-weather activities, but has negative impacts on cold-weather activities, such as alpine skiing, snowmobiling, polar bear sighting and glacier viewing [2]. In Countries with a temperate to hot climate, climate change and the increased temperatures can significantly affect the number of visits to nature parks. For example, Fisichelli *et al*., assessed the relationship between climate and park visitation in the USA, by correlating historical (1979–2013) visits to temperatures [3]. They found more visits in months with average temperatures below 25°C, and a strong decrease in visits when temperatures were above 25°C. Similar results were found in a study that assessed the relationship between outdoor temperature and number of visits to Chester Zoo, England. The researchers found a steady increase in the number of visits up to a threshold of 21°C, but a decrease in the number of visits in hotter days [4]. Similarly, Clodrey and Turpie (2000) developed models to understand the effect of temperature on tourist visits to South Africa’s 19 national parks. They suggested that the continuous changes in climate change (namely, the increase in average temperatures) may decrease the number of visits in the future. Using the models, the authors predicted significant declines in the number of visits to some of the national parks, due to the increase in outdoor temperatures [5]. Hiking or outdoor visits on hot days may also expose the visitors to physical discomforts (for example due to sweating), but also to health risks such as dehydration, heat exhaustion or hyponatremia [6]. Moreover, exposure to high temperatures and heatwaves is also associated with adverse health effects, such as cardiovascular diseases, and can even lead to deaths [7, 8].

An additional factor that may potentially affect the number of visits to natural parks and reserves, is air pollution, which is also related to climate change: emissions of pollutants into the air can result in changes to the climate, on the other hand, climate change has the potential to increase air pollutants, such as ground-level ozone [9]. Recent studies have suggested two possible mechanisms by which air pollution negatively affects visitation frequency to nature parks. First, air quality warnings (focusing on adverse health effects due to exposure to air pollution) that are published on days with poor air quality, may deter visitors from frequenting parks, and second, visitors may decrease visitation frequency on days with poor visibility, and air pollutants may contribute to such reduced visibility. Exposure to air pollutants is associated with a variety of adverse health outcomes: respiratory diseases, cardio-vascular disease, diabetes, obesity and even various types of cancer [10, 11]. However, air pollutants are still found in high concentrations globally, and the population is exposed to these pollutants [12].

Few studies so far aimed to estimate the relationship between air pollution and visits to natural parks. Keiser *et al*. compared ozone concentrations and number of visits in US national parks and found a robust negative association: higher concentrations were associated with fewer visits [13].

Jiang *et al*. showed that particulate pollution has a negative impact on the number of visitors in parks in Beijing, China. Furthermore, they found that when pollution levels changed from moderate to heavy pollution (PM_2.5_ concentrations higher than 150 (mg/m^3^)), there was a significant drop in the number of visits [14].

In this study, we evaluated the effect of both air pollution exceedance and heatwaves, on the number of visitors in national parks and nature reserves with paid entrance in Israel. We concentrated on ambient particulate matter, 10μm in diameter or smaller (PM_10_), as an indicator of air pollution, and on heatwave events (three consecutive days with maximum temperature of ≥32.2°C in central Israel, following the Israel Meteorological Service definitions) and compared the number of visits between days with/without air pollution exceedances and days with/without heatwaves, on a national and local scale.

## Methods

### Data

Data of local visitor entries was collected from 56 national parks and nature reserves in Israel during the years 2016–2019, in total, 75,165 records on visitor entries, by site and date. Of note, five small, less visited remote national parks and reserves were removed from the analysis due to inconsistent visit patterns. Entries with missing data (days with no available data regarding the number of visits at specific sites) were omitted, and the final database was comprised of 68,518 visitor entries from 51 parks and national reserves.

We focused on several environmental indicators: maximum daily temperatures, heatwaves, and air pollution levels: The maximum temperature for each entry (defined as the specific combination of site and date) was retrieved from the Israel Meteorological Service (IMS) [15], using the data for the nearest IMS station. National heatwaves were defined as three consecutive days with a maximum temperature 32.2°C in central Israel (Bet Dagan monitoring station), based on the IMS. The definition of three consecutive days with maximum temperatures of ≥32.2°C is commonly used around the world and was also adopted by the Israeli Ministry of Health [16].

To reflect air pollution levels, we focused on levels of ambient particulate matter (PM), 10μm diameter or smaller (PM10). Due to the non-uniform spatial distribution of monitoring stations in Israel, and the fact that some of the monitoring stations are far from the sites included in the study, we decided to focus on PM10 levels in monitoring stations that are adjacent to Israel’s biggest cities, which cover 51% of the Israeli population. This was also done to ensure coverage of pollution levels at the visitors’ departure locations, to reflect the possibility that people choose whether to visit a park based on the pollution level at their residential address (*i*.*e*. departure location). In order to define days with a national PM10 exceedance, we used the Israeli environmental PM10 values—concentrations higher than 130°g/m^3^ [17]. PM10 level, and exceedances in these levels were retrieved from Air monitoring in Israel Ministry of Environmental Protection [18], (see S1 Table).

### Statistical tests

In order to analyze the effect of heatwaves and exceedances in PM10 pollution levels on the number of visitor entries to national parks and nature reserves, and in order to rule out any potential bias due to visitor overload during holidays and weekends, we performed several statistical tests in which only nonholiday periods were examined. We used nonparametric tests due to the non-normal distribution of the visitors’ data.

Pearson and Spearman correlations were used to examine associations between number of visits and ambient temperatures, according to geographical (whole country, or per district), and temporal (annual or seasonal) categories, and whether sites are with/without a water source (beach, river, etc.).

Linear and quadratic multiple linear regressions were used to investigate associations between temperature, air pollution (exceedance in PM10), wars (military operations), and number of visits, in the various geographical and temporal categories. The explaining variable in the linear model were the temperature, PM10, and wars, while the explaining variables in the quadratic models were temperature (linear component), temperature^2^ (quadratic component), PM10, and wars. The logarithm value of the number of visits was used in all multivariate linear regressions.

The multivariate linear regressions formulas were as following:

Linear models: log($\bar{Y}$) = *β*_0_ + *β*_1_ * *temperature* + *β*_2_ * PM_10_ + *β*_3_ * *wars*

Quadratic models: log($\bar{Y}$) = *β*_0_ + *β*_1_ * *temperature* + *β*_2_ * *temperature*^2^ + *β*_3_ * PM_10_ + *β*_4_ * *wars*

Where:

$\bar{Y}$ = estimsted number of daily visitors

*β*_0_ = intercept

*β*_1,_*β*_2,_*β*_3,_*β*_4-_regression coefficients

temperature–maximum daily temperature

PM10—Exceedance in PM10

wars–occurrence of war / military operation on that day

In the linear regression analyses, all explaining variables were normalized using max-min normalization, in order to understand the relative contribution of each variable. To further evaluate the significance of the two main parameters (temperature and PM10 exceedance), we conducted additional linear regressions excluding these variables. The following required assumption for running linear regressions were validated: there were no dependencies between the observations, there was no multicollinearity between the independent variables, numerical values followed a normal distribution, and there were no influential outliers.

One-sided Wilcoxson paired tests were used to evaluate the effect of national air pollution on the number of visits: (a) to compare the number of visits on days with pollution exceedances to the number of visits in the following days with no exceedances (total of 1,610 paired days), and (b) to compare the number of visits on days with no pollution exceedances to the number of visits in the following days (with pollution exceedances), (total of 1,497 paired days). For multiple comparisons, we used FDR correction.

In order to study the effect of heatwaves on the number of visits (in all studied sites), and to avoid the comparison of weekends and non-weekend days, we used one-sided Wilcoxson paired tests, to compare the number of visits in weeks with heatwaves (at least three consecutive days in which the temperature in central Israel (Bet Dagan monitoring station) was over 32.2°C)), to the following weeks (with no heatwaves), a total of 288 paired weeks (rather than comparison of heatwaves days to the consecutive non-heatwave days).

In addition, we used one-sided Wilcoxson tests to compare the average number of visits in each site between days within heatwaves (one of at least three consecutive days with temperatures of 32.2°C or higher, in central Israel) and days with no heatwaves, and the number of visits in each site between days with PM10 exceedances and days with no such exceedances, using both the national exceedance. We also used Fisher’s exact test to compare between sites in which there were more/less visits in heatwaves and whether the site is a nature water reserve/park (with a river/beach/lake, etc.).

Finally, we used Fisher’s exact test to compare the occurrence of heatwave days (one of at least three consecutive days with temperatures 32.2°C or higher, in central Israel) and PM10 pollution levels in order to examine whether heatwaves are associated with air pollution.

All statistical analyses were performed using Python (version 3.9.13).

## Results

### Associations between temperature and number of visits

**Fig 1:**
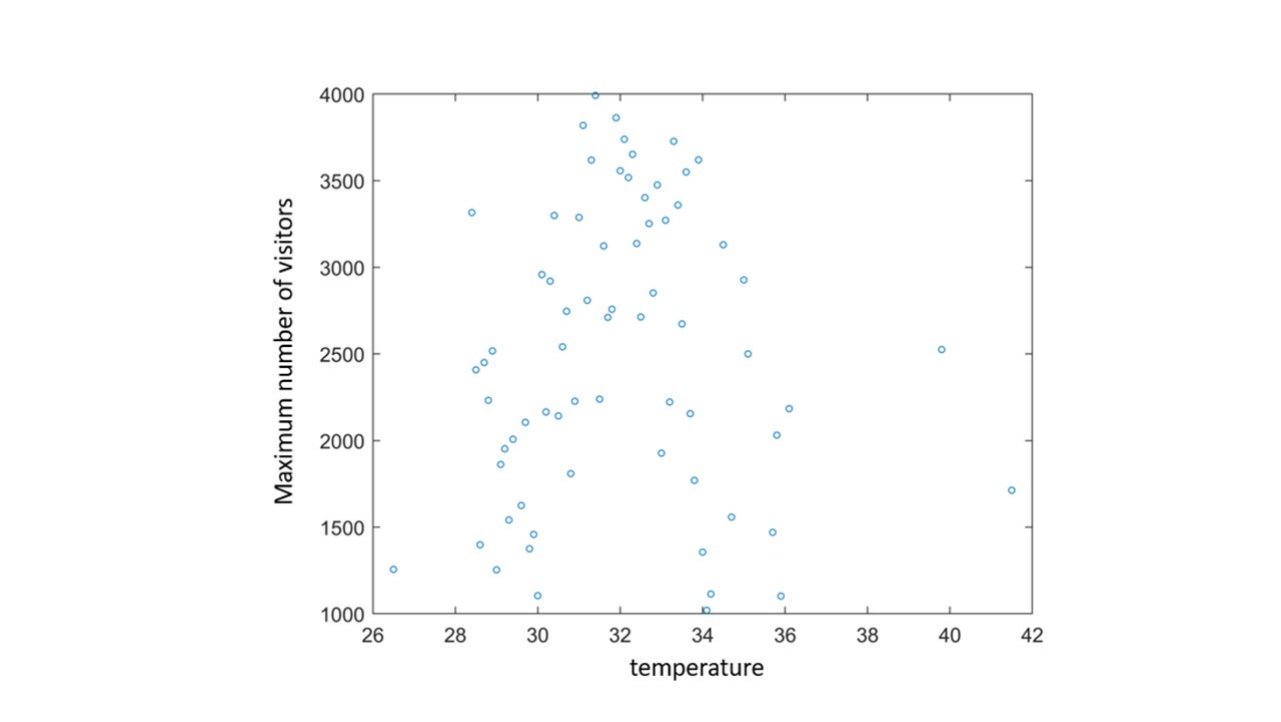
Maximum number of visitors in summer days as a function of temperature. X axis represents temperature and Y axis represents the maximum number of visits.

There was no significant linear correlation between temperature and number of visits in all sites when considering all sites (not shown). To further investigate the nature of associations between temperature and number of visits, we used linear regressions. In most models (7 out of 8), the temperature component was statistically significantly positive, meaning that higher temperatures were associated with more visits. Of note, the temperature coefficient was negative in the southern sites, in which the annual temperatures are usually higher than other sites. Furthermore, the quadratic models exhibited lower p-values compared to the linear models, suggesting their superior performance. To investigate associations which may not necessarily be linear (but unimodal, for example), we used quadratic linear regressions. In all quadratic models, the quadratic component (temperature^2^) coefficients were negative, meaning that a higher number of visits occurred on milder days compared to hotter or colder ones, as can be seen in Fig 1 and in Table 1. PM10 exceedances were statistically significantly negatively associated with the number of visits in most of the models, meaning that there were fewer visits in polluted days. As expected, military operations were statistically significantly negatively associated with the number of visits in almost all models.

**Table 1:** Linear and quadratic regressions for the association between temperature, PM10, wars, and number of visits.

District	Type of model (degree of temperature component)	Intercept (p-value)	Temperature linear component beta (p-value)	Temperature quadratic component beta (p-value)	Exceedance in PM10	War / military operation	Model RMSE (p-value)
National	Linear	3.76 (<0.001)	1.36 (<0.001)	-	-0.11 (0.001)	-0.28 (<0.001)	1.66 (1.6e-197)
	Quadratic	1.72 (<0.001)	11.29 (<0.001)	-9.29 (<0.001)	-0.06 (0.09)	-0.08 (0.04)	1.65 (7.1e-316)
North	Linear	3.36 (<0.001)	1.99 (<0.001)	-	-0.13 (0.002)	-0.25 (<0.001)	1.63 (3.5e-298)
	Quadratic	1.92 (<0.001)	11.5 (<0.001)	-9.8 (<0.001)	-0.08 (0.07)	-0.05 (0.32)	1.62 (0)
Central	Linear	4.49 (<0.001)	1.21 (<0.001)		-0.28 (0.001)	-0.49 (<0.001)	1.36 (2.4e-45)
	Quadratic	3.22 (<0.001)	11.1 (<0.001)	-10.2 (<0.001)	-0.21 (0.01)	-0.35 (0.0002)	1.35 (9.9e-71)
South	Linear	5.1 (<0.001)	-0.79 (<0.001)	-	0.006 (0.9)	-0.19 (0.03)	1.85 (3.2e-9)
	Quadratic	3.24 (<0.001)	8.41 (<0.001)	-8.69 (<0.001)	0.05 (0.51)	0.009 (0.92)	1.84 (1.3e-27)

To further evaluate the association between the two main parameters (temperature and PM10 exceedance) and the number of visits, we conducted additional linear regressions excluding these variables. The positive contribution of the temperature and the negative contribution of both PM10 and wars were robust and were found in all models. Comparison between the three models (all parameter, no temperature and no PM10), emphasizes the significant contribution of both parameters (temperature PM10) to the prediction of visits. As expected, the temperature parameter contributes more to the prediction of number of visits, in comparison to the PM10 parameter. (See Table 2).

**Table 2:** Comparison of multivariate quadratic regressions with and without temperature and PM10, (national visits).

Type of model	Intercept (p-value)	Temperature linear component beta (p-value)	Temperature quadratic component beta (p-value)	Exceedance in PM10	War / military operation	Model RMSE (p-value)
All parameters	1.72 (<0.001)	11.29 (<0.001)	-9.29 (<0.001)	-0.06 (0.09)	-0.08 (0.04)	1.65 (7.1e-316)
No temperature	4.5 (<0.001)	-	-	-0.1 (0.007)	-0.5 (<0.001)	1.67 (4.6e-40)
No exceedance in PM10	1.71 (<0.001)1	11.3 (<0.001)	-9.33 (<0.001)	-	-0.1 (0.02)	1.65 (1.1e-310)

**Fig 2:**
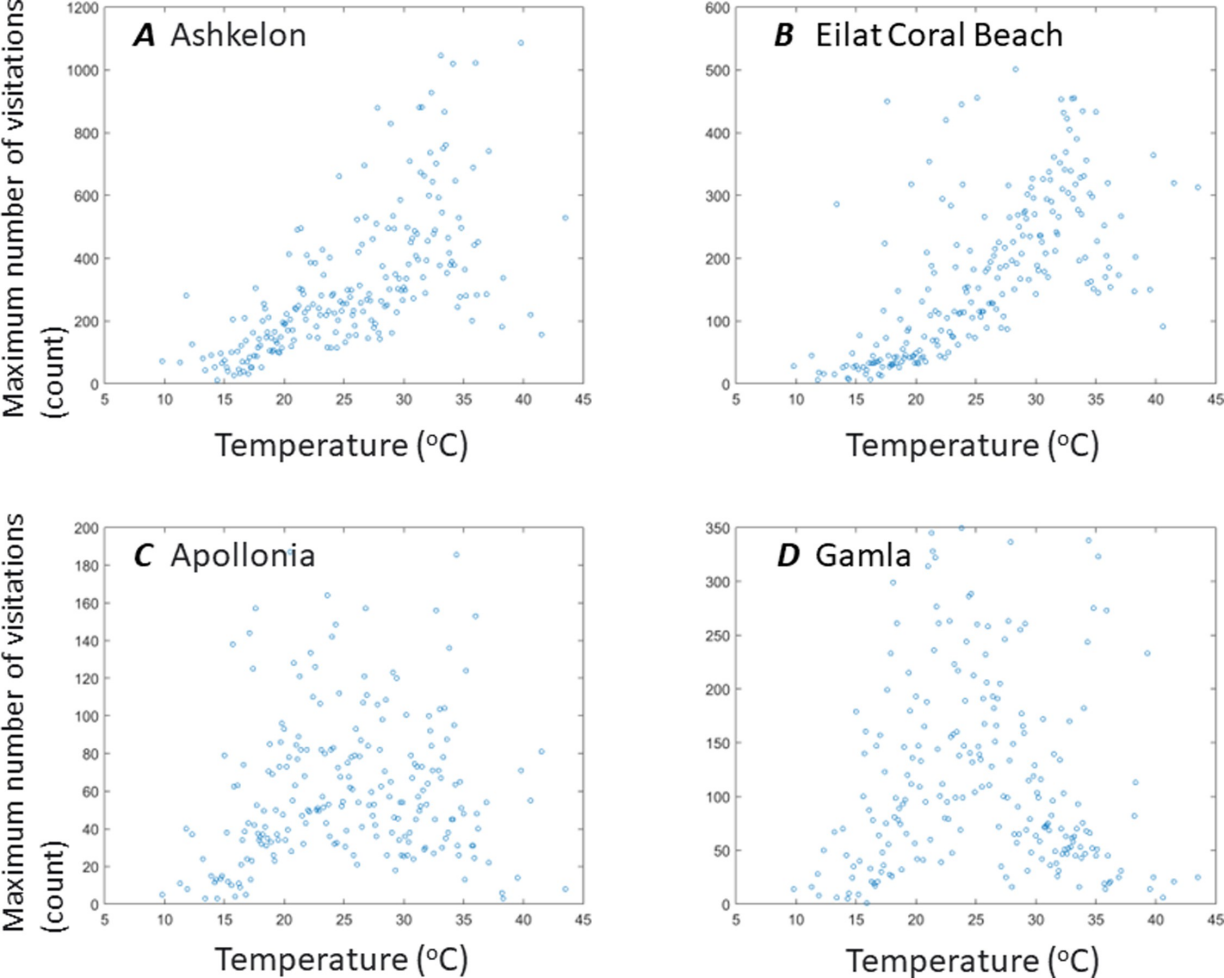
Median number of visitors in selected sites as a function of temperature. X axis represents temperature and Y axis represents the median number of visits in days at a specific recorded temperature. Sites with beaches (A: Ashkelon and B: Eilat Coral Beach) and sites with no beach or other types of water sources (C: Appolonia and D: Gamla).

A more specific examination of the different sites (on a site resolution) reveals that the association between the number of visits as a function of temperature, both in sites with beaches and in sites with no beaches, follows a unimodal distribution (as in the national analysis). In addition to the unimodality, positive linear correlations were found between temperature and number of visits on all sites with beaches (in addition to the unimodal distribution): The Akhziv site (r = 0.43, p<0.001), the Ashkelon National Park (r = 0.56, p<0.001), the Dor HaBonim Beach (r = 0.52, p<0.001), and the Eilat Coral Beach (r = 0.6, p<0.001), probably due to a more asymmetric nature of the unimodal distribution. See also Fig 2.

### Effect of heatwaves on the number of visits

Of the 1095 days (more than four years, not including weekends) between 2016–2019, 202 days were considered part of a heatwave event. The weekly average national number of visitors in all sites combined, was significantly lower in weeks with heatwaves (median of 1648 ± 44.7 visitors per week) compared to weeks with no heatwaves (median of 2018 ± 47.9 visitors per week), (Wilcoxon test, p < 0.001)–a median decrease of 18.3%.

**Fig 3:**
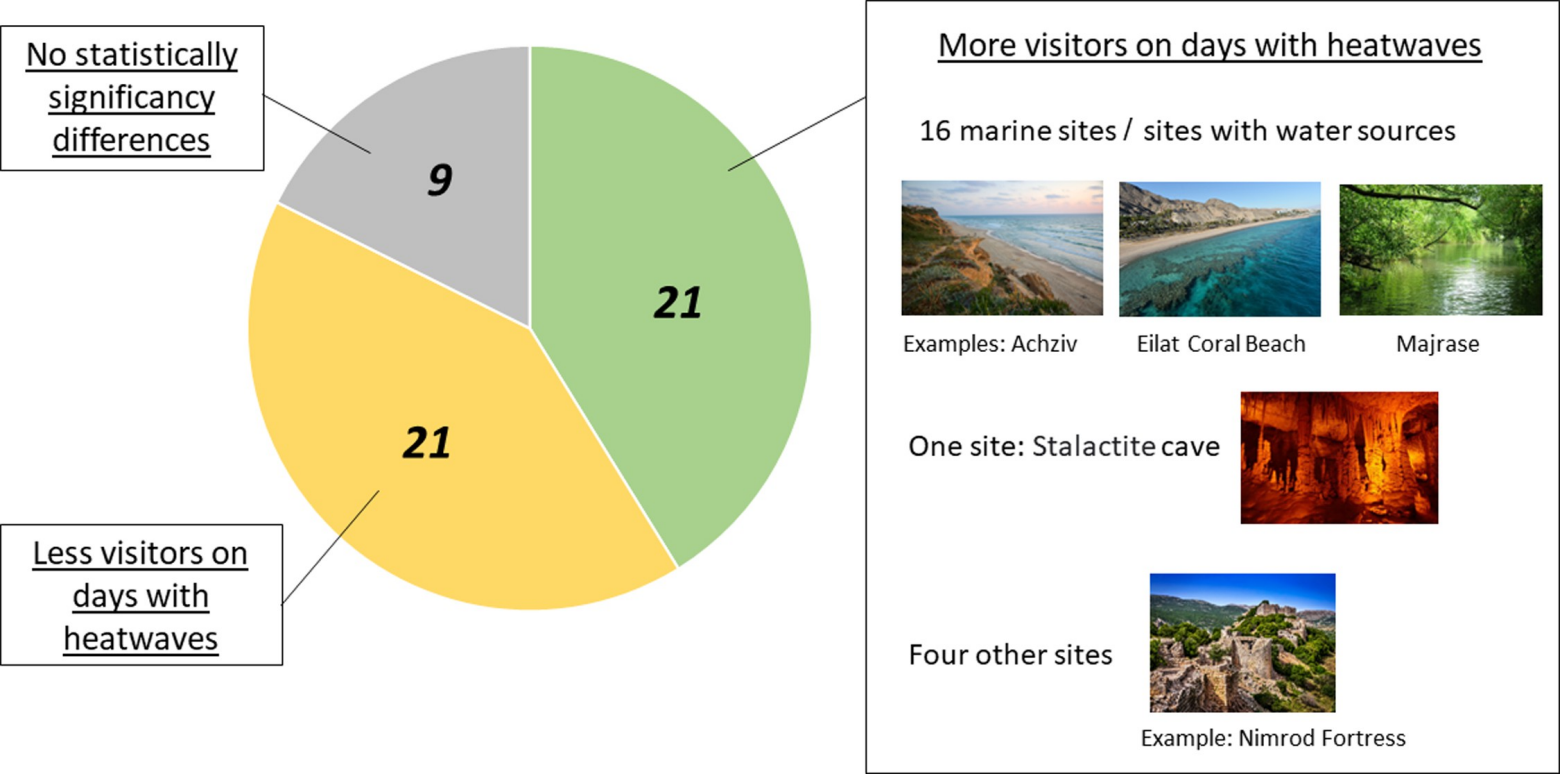
Increase and decrese in the number of visits in heatwaves.

Next, we compared the average number of visitors in each site, between days with heatwaves and days without heatwaves. Out of the 51 examined sites, statistically significant differences were found in 42 sites. In 21 of these 42 sites, the numbers of visits on days with heatwaves were significantly lower than the number of visits in days with no heatwaves (p<0.05, Wilcoxon test). Examination of the remaining 21 sites, in which the number of visits in heatwave days was significantly higher than the number of visits in days with no heatwaves (p<0.05, Wilcoxon test), reveals that most of these sites (16/21) have water sources (beaches, lakes, rivers etc), that enable aquatic activities, and one site is a wide cave site (Stalactite Cave Nature Reserve) with a stable temperature of 22°C, not affected by outside temperatures (See Fig 3). Indeed, a comparison of the associations between number of visits during heatwaves and whether the site contains a water source yielded statistical significance (Fisher exact test p<0.001), meaning that during heatwaves, there were relatively more visitors to sites with a water source.

### Effect of air pollution on the number of visits

Of the 1095 days, there were 62 days with a national exceedance of PM10 concentrations. There was a statistically significant association between days within heatwaves and PM10 exceedances (Fisher test p<0.001).

The average number of visitors in all sites combined, in days with national PM10 exceedances (mean ± standard deviation of 240 ± 90 visitors), was statistically significantly lower than these numbers in days after without exceedances (mean of 273 ± 110), (Wilcoxon test p < 0.001). The average number of visitors in non-polluted days (days with no exceedances in national PM10), (309 ± 116 visitors) was statistically significantly higher than the number of visitors in days after without such exceedances (252 ± 95 visitors), (Wilcoxon test, p < 0.001).

Next, we compared the average number of visitors in each site between days with national PM10 exceedances and days without such exceedances. Out of the 51 examined sites, there were statistically significant differences in seven sites (and in four sites after FDR correction). In all these sites, the numbers of visits in days with PM10 exceedances were statistically significantly smaller than the number of visits in days with no such exceedances (p<0.05).

## Discussion and conclusions

Climate changes are manifested in rising global temperatures, more frequent and intense heatwaves, and other meteorological phenomena, all of which have wide implications on human health and behavior. Tourism is considered as a climate change—vulnerable sector, influenced by cold, heatwaves, and additional meteorological phenomena.

In this study we examined how climate change related events, such as heatwaves and exceedances in air pollution, affect touristic activity, in particular visits to natural parks and reserves in Israel, using a database of visits from the years 2016–2019. Of note, we did not analyze visits after 2019 due to the COVID-19 pandemic that led to significant changes in visitation patterns of the public, and regulations of the Israel Nature and parks authority.

Our major finding revealed that heatwave events are characterized by an overall lower number of visitors to national parks and nature reserves. Interestingly, at the site level, the effect of heatwaves on visitor numbers was attenuated by the existence of a water source at the site. This finding is consistent with previous works that showed changes in leisure time behavior during heatwaves, for example Gómez-Martín *et al*. showed an increase in visiting to beaches during heatwaves in Spain [19]. We found that the number of visits as a function of temperature follows a unimodal distribution, meaning that there are more visits in days with moderate temperatures, in comparison to extremely hot or cold days.

We found a significant negative association between air pollution (days with exceedance in PM10) and number of visits, using both multiple linear regression, and statistical inference. On weeks with exceedances in air pollution levels, fewer visitors were recorded compared to weeks with no exceedances in air pollution levels. Although the association was statistically significant, we cannot rule out the possibility that the reduction in the number of visits was not caused directly by air pollution, but by other factors. Indeed, a significant association between days within heatwaves and PM10 exceedances raises the possibility that the two variables are confounded. The association between heatwaves and PM10 exceedances was found in previous works, for example, Kalisa *et al*. found a positive correlation between temperature and PM10 levels in Birmingham, UK [20]. Future studies in Israel should examine the effect of air pollution on visits to national parks and reserves.

Our study has two major limitations: (a) The data refers to visits in general, with no specific information regarding the individual visitors (age, gender), purpose of visit (hiking, camping, religious purposes), or visit duration, which could fine tune the findings and shed additional light on the effect of heatwaves, (b) Our study did not take into consideration the possible effect of the time of day in which the visits took place, which may influence visiting patterns. For example, in hotter days, people may tend to visit more in evening hours and less in the mornings/afternoons.

On the other hand, the study has some significant strengths: (a) This big data study is based on detailed visitor records collected during four full years, (b) Since the entrance to the sites is paid, entrance to the sites is regulated, and the data collected is therefore highly reliable, and (c) The study is highly relevant to a large number of countries, with an emphasis on areas with rising temperatures, and higher frequencies of heatwaves.

As climate change worsens, temperatures increase globally and in Israel, leading to higher frequencies of heatwaves [21, 22], we expect, based on the findings of this study and others, to see a decrease in the number of visits to national parks during heatwaves (particularly those with no water sources). This decrease can have serious implications in the long run, for example on the public’s choice of leisurely activities, and may harm the connection of the public with nature, heritage, and history. In addition, heatwaves may lead to shift in the distribution of visit frequencies in favor of sites with water sources, and specifically sites with beaches. We suggest that nature protection authorities should take these findings into consideration as part of their adaptation strategies to heatwaves in specific and climate changes in general.

In addition to the importance of actions to reduce carbon emissions and combat climate change, this study also emphasizes the need to improve and create better adaptation strategies to climate changes in natural reserves and parks. Some of the solutions may be easy and readily feasible, for example, installing more shadings and water coolers in national parks and reserves, while other solutions should be examined more carefully, while integrating other aspects of nature parks. For example: enabling night visits (that may disturb the activity of nocturnal animals and may result in light pollution), planting trees for shading (that may not fit the ecological systems), construction of artificial ponds or small lakes, etc.

In conclusion, this study sheds light on another significant impact of climate change—leisure activities. The study emphasizes the need for increased awareness and readiness regarding the effect of heatwaves on visits to nature reserves and parks.

## Supporting information

S1 Table Data variables (features) collected per site and date. (DOCX)

S2 Table Representative data (Minimal data set). (XLSX)
