# Supplementary material for: The effect of heatwaves on the number of visits to national parks and reserves
Source: PLoS One. 2023 Aug 9;18(8):e0289201. doi: 10.1371/journal.pone.0289201 (PMC10411752; doi:10.1371/journal.pone.0289201)
Supplement: S1 Table — (DOCX) [file pone.0289201.s001.docx]

The effect of heatwaves and air pollution on the number of visits to national parks and reserves

Supplementary material

**Supplementary table 1 - data variables (features) collected per site and date**

| **Feature type** | **Feature** | **Variable type** |
| --- | --- | --- |
| Site characteristics | Region (Center, north, south) | categorical |
|  | Site contains water source? | Binary |
| Date characteristics | If the date is on weekend | Binary |
|  | If the date is a holiday | Binary |
|  | If there was a war / military operation on that day | Binary |
| Environment | Exceedance in national PM_10_ level | Binary |
|  | Temperature on this date (in Celsius) | Numeric |
|  | If this date is defined as a heat wave | Binary |
